# Quantifying dislocation-type defects in post irradiation examination via transfer learning

**DOI:** 10.1038/s41598-025-00238-5

**Published:** 2025-05-07

**Authors:** Michael Wu, Jeremy Sharapov, Matthew Anderson, Yu Lu, Yaqiao Wu

**Affiliations:** 1 Idaho National Laboratory, Idaho Falls, ID USA; 2 Boise State University, Boise, ID USA; 3 Center for Advanced Energy Studies, Idaho Falls, ID USA

**Keywords:** Machine learning, Dislocation defect quantification, Post irradiation examination, Metals and alloys, Nuclear energy

## Abstract

The quantitative analysis of dislocation-type defects in irradiated materials is critical to materials characterization in the nuclear energy industry. The conventional approach of an instrument scientist manually identifying any dislocation defects is both time-consuming and subjective, thereby potentially introducing inconsistencies in the quantification. This work approaches dislocation-type defect identification and segmentation using a standard open-source computer vision model, YOLO11, that leverages transfer learning to create a highly effective dislocation defect quantification tool while using only a minimal number of annotated micrographs for training. This model demonstrates the ability to segment both dislocation lines and loops concurrently in micrographs with high pixel noise levels and on two alloys not represented in the training set. Inference of dislocation defects using transmission electron microscopy on three different irradiated alloys relevant to the nuclear energy industry are examined in this work with widely varying pixel noise levels and with completely unrelated composition and dislocation formations for practical post irradiation examination analysis. Code and models are available at https://github.com/idaholab/PANDA.

## Introduction

Understanding the effect of radiation on a material’s properties and performance is crucial for safe and reliable nuclear energy operations. Dislocation lines and loops are common defects induced during irradiation which significantly affect the properties and durability of materials^1^. Quantifying dislocation defects is necessary to relate material performance to the effects of radiation and this is often done via transmission electron microscopy (TEM) where the dislocation defects are manually identified in the micrograph by an instrument scientist. However, manually identifying dislocation defects in micrographs results in a defect quantification that can be error-prone, and lack consistency and reproducibility. To improve consistency and reproducibility, earlier studies have used the public domain software ImageJ to label defects^2–4^, where microscopy images went through a preprocessing step based on contrast to quantify defects. More recently, however, developing consistent and reproducible automated methods using machine learning to obtain the total number and distribution of defects, such as grain boundaries, precipitates, and dislocation lines and loops, has drawn great interest in the material science community^5–12^. These approaches provide the possibility of analyzing TEM micrographs consistently and much faster than the conventional approach outlined in Nathaniel et al.^13^. These machine learning approaches are fast enough to be used for in-situ TEM measurements where the defects can evolve in real time.

A wide variety of machine learning approaches have been applied to identifying dislocation defects in TEM micrographs. One of the first attempts to use machine learning to classify defects using machine learning was by Masci et al.^5^ where a max-pooling Convolutional Neural Network (CNN) approach was developed to classify steel defects. Later on, Li et al.^6^ combined different computer vision techniques to detect a specific sub-class of dislocation loops in a pipelined workflow. Their CNN model was trained on 1605 augmented images that had been labeled with bounding boxes around the defects. The model reported both precision and recall greater than 0.8 on the six images in the selected test dataset. The images, however, contained very little noise , which does not generally represent the types of micrographs that an instrument scientist works with. Later, Shen et al.^7^ used a faster Regional CNN (R-CNN) for defect detection to show the performance improvement compared to human analysis. This model was trained on 918 augmented images and obtained precision and recall ranging from 0.62 to 0.83. In the subsequent year, Jacobs et al.^12^ expanded on the model introduced by Shen et al.^7^ by introducing semantic segmentation of multiple defect types for the same FeCrAl alloys using Mask R-CNN model implemented using the Detectron2 package. In a separate work, Shen et al.^8^ utilized a YOLOv3 bounding box detection system to automate TEM video analysis for microstructural features, where the model was trained on 12 frames with real-time augmentation and 18,300 epochs plus transfer learning. Roberts et al.^9^ present DefectSegNet, a novel hybrid CNN model for robust and automated semantic segmentation of dislocation lines, precipitates, and voids, trained on 48 augmented images. To address the systemic issue of the lack of training data, Govind et al.^10^ proposed the potential of synthetic data in overcoming the limitations of the manual annotation of dislocations by using a U-Net++ model trained with 4000 synthetic images. Most recently, Bilyk et al.^11^ trained a Mask R-CNN model on 32,977 augmented images on micrographs of Y3-Fe, Y3-Ni, and ES1-Ni to predict on three different types of dislocation loop classes. One such augmentation was adding noise, and as such this model was able to predict on noisier images compared to previous studies.

This work complements those efforts by focusing on high noise micrographs representative of real-world conditions. Additionally, the approach explored here substantially reduces the training dataset size to just five TEM defect annotated images, and does not require any post-processing on the predictions. There is a pressing need for highly accurate models trained on small datasets^14^ due in part to the difficulty and expertise required to correctly annotate defects in micrographs. This work uses YOLO11 (You Only Look Once version 11)^15,16^, a state-of-the-art object detection framework that builds upon the YOLOv8 framework, which is known for its versatility, effectiveness, and popularity in real-time image analysis tasks with a user-friendly interface^17^ to explore automated post irradiation examination defect quantification. YOLO models are often used in conjunction with transfer learning approaches and this has been key to the success of YOLO^18^.

Three different alloys are explored in this work: MA956 ODS, FeCrAl, and HfAl alloy. The MA956 ODS alloy is a commercial oxygen dispersion-strengthened (ODS) alloy with superior oxidation resistance and creep strength at elevated temperatures, and is considered a promising cladding material that can withstand ultra-severe environments for the next generation of advanced nuclear reactors^1,19,20^. The FeCrAl alloy is also known for superior temperature, radiation, and corrosion resistance, and has applications in nuclear fuel cladding^21^. HfAl shows good mechanical strength at high-temperatures^22^. The TEM micrographs for MA956 ODS and HfAl used in this study are available via the Nuclear Science User Facilities’ Nuclear Research Data System^23,24^ while the FeCrAl micrographs are from Shen et al.^7^.

This work is structured as follows. The training, validation, and test datasets are described in “Methods”, where the machine learning models and training procedure are also described. In “Results”, the model predictions are quantitatively evaluated on test datasets that include all three alloys and include both noisy and non-noisy micrographs. In “Discussion”, the model is discussed and compared with other machine learning approaches. In “Conclusion”, the conclusions are presented and future work is discussed.

## Methods

Two transfer learning models denoted model A and B were trained for this study. The training datasets for these two models were different to reflect different transfer learning strategies. Both models were trained and validated on a total of five MA956 ODS alloy images. These images were then segmented into 50 images, where 45 were used for training and five were used for validation. Both models started training using pre-trained segmented MSCOCO weights^25^ from Ultralytics. Apart from the pre-trained MSCOCO weights and the MA956 ODS dataset, no other data was used to train model A.

Training data for model B was augmented beyond what was used for model A by adding 41 images of a crops dataset^26^ and 53 images of synthetic cavities.^27^ The use of the crops and synthetic cavity datasets was driven by their resemblance to dislocation lines and loops respectively. These datasets have a structural similarity index (SSIM)^28^ of on average 0.25 compared with the MA956 training images where SSIM assesses image similarity between −1 (dissimilar) and 1 (similar). Some MA956 images have SSIM approaching 0.4 with the crops and synthetic cavities datasets. Model B is a transfer learning approach that leverages augmented datasets that only have a moderate structural similarity to the MA956 training images.

To evaluate the quality of the two models, the test dataset consisted of a total of four images that were not included in either the training or validation datasets. The test dataset images include two MA956 ODS images, one from Shen et al.’s FeCrAl dataset, and one image of HfAl. Figures 2, 3, 4, 5, and 6 show the test dataset images.

For the MA956 ODS alloy and HfAl micrographs, TEM characterization was carried out at the Microscopy and Characterization Suite (MaCS), in the Center for Advanced Energy Studies (CAES). A ThermoFisher (FEI) 3D Quanta Focus Ion Beam (FIB) was used to lift out TEM lamellae. To minimize the artifacts from FIBing, a low ion accelerating voltage (2 kV) was used in the final thinning step and the TEM lamellae was cleaned after FIBing using a Fischione Model 1040 Nanomill. TEM lamellae were characterized with a ThermoFisher (FEI) Tecnai G2 F30 Scanning TEM (STEM). The on-zone axis Bright-Field Scanning TEM (BFSTEM) imaging^29^ was used to show all the irradiation induced defects including dislocation loops and dislocation lines.

### Machine learning model

This study uses the YOLO11x segmentation model from Ultralytics. YOLO11 is the 2024 version of the You Only Look Once (YOLO) model first released in 2015. Compared to previous YOLO models such as YOLOv8, YOLO11 enhances inference speed and accuracy on test datasets while featuring 22$\%$ less parameters compared with similar YOLOv8 models^16^. Additionally, for segmentation tasks, YOLO11 can segment individual features down to the pixel level, which is invaluable for segmentation tasks that need to be precise^15^, such as dislocation identification.

**Fig. 1:**
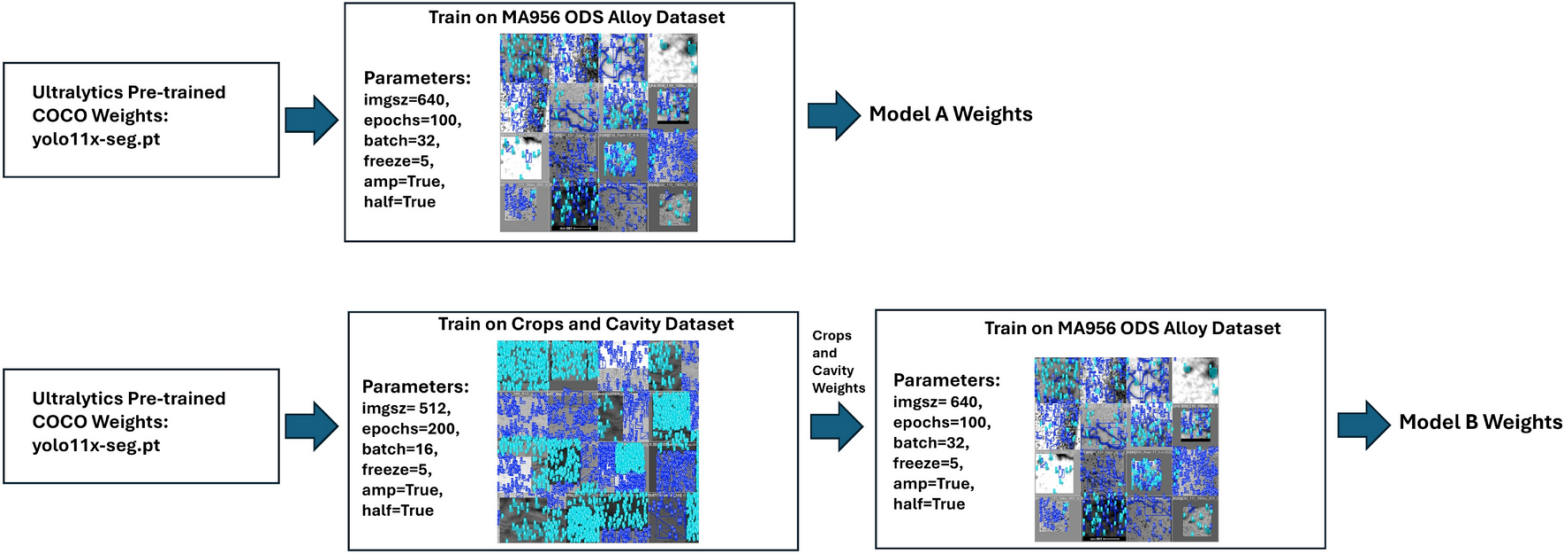
An overview of the transfer learning training process for dislocation defect quantification is summarized here. Two transfer learning models are evaluated in this work and both begin with pre-trained MSCOCO weights. Using the pre-trained weights, model A is trained on a dataset consisting only of MA956 ODS micrographs. In contrast, model B is trained on the crops^26^ and synthetic cavity^27^ datasets. The best weights from this intermediate stage are then used to train on MA956 ODS training images which produce the weights of model B.

The training process for models A and B is shown in Fig. 1. Model A starts with the pre-trained MSCOCO weights and trains on the MA956 ODS alloy images for 100 epochs. Model B starts with the pre-trained MSCOCO weights and trains on the crops and cavity image database for 200 epochs. This intermediate result produces the crop and cavity model weights. The best weights from crop and cavity model, which are the weights from the epoch with the highest validation metrics, are then used to train model B on the MA956 ODS alloy images for 100 epochs. Key YOLO11 parameters for tuning the models are summarized in Table 1. A detailed description of the parameters can be found at Ultralytics^16^. The full code and models, along with the training, test, and validation datasets, are available at https://github.com/idaholab/PANDA.

**Table 1:** Key parameters used for fine-tuning the models. *imgsz* indicates the dimensions all images are resized to when training the model; *epochs* denotes the total number of complete passes over the full dataset for training; *batch* specifies the number of training images analyzed before updating the model’s weights; *freeze* determines the number of initial layers to be frozen with the weights remaining unchanged; *amp* enables automatic mixed precision; and *half* enables half precision inference.

Model	imgsz	epochs	batch	freeze	amp	half
Model A	640	100	32	5	True	True
Crops and Cavity	512	200	16	5	True	True
Model B	640	100	32	5	True	True

## Results

In this section, inference results from the two different transfer learning strategies denoted by models A and B are presented. The precision, recall, and F1 score are given for each inference case. The metrics of precision, recall, and F1 score for each test image inference can be viewed in Table 2, and an estimate for the amount of noise in each image, computed by the standard deviation of the pixel values^30^, is also given under each image. Note that the YOLO11-based models allow for overlapping prediction masks, resulting in teal segments in the predictions for areas where a dislocation loop and dislocation line mask intersect.

**Fig. 2:**
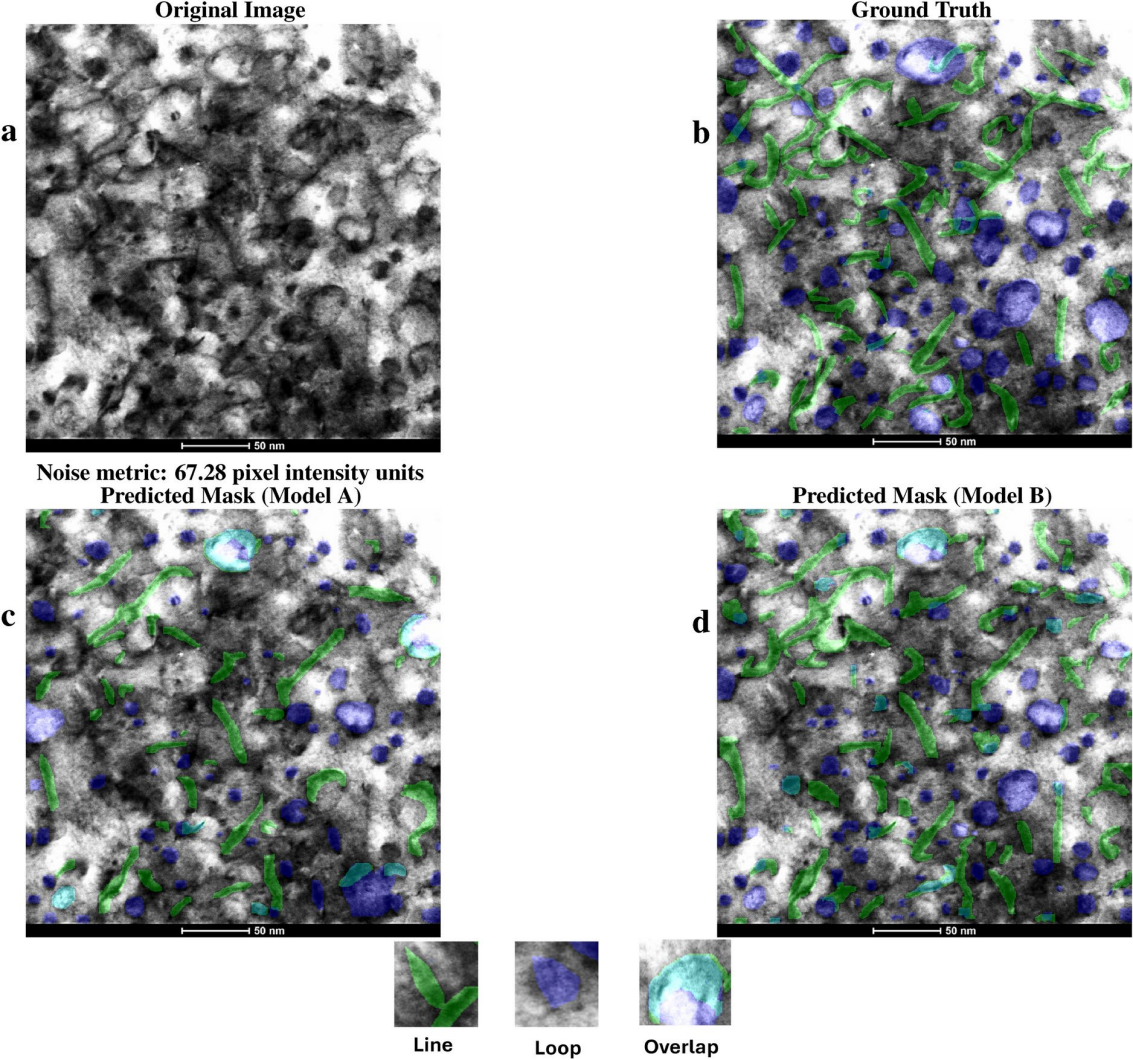
Comparison between the original image (**a**), ground truth labels (**b**), model A predictions (**c**), and model B predictions (**d**) of a Bright Field STEM image taken under the [001] zone axis with 380kx magnification from the MA956 ODS alloy that is Fe^2+^ irradiated to 25 dpa at $320^\circ$C. Green masks show lines, blue masks show loops, and teal shows overlapping predictions of both lines and loops. Model B identifies more lines and has more accurate loop placement compared to model A.

**Fig. 3:**
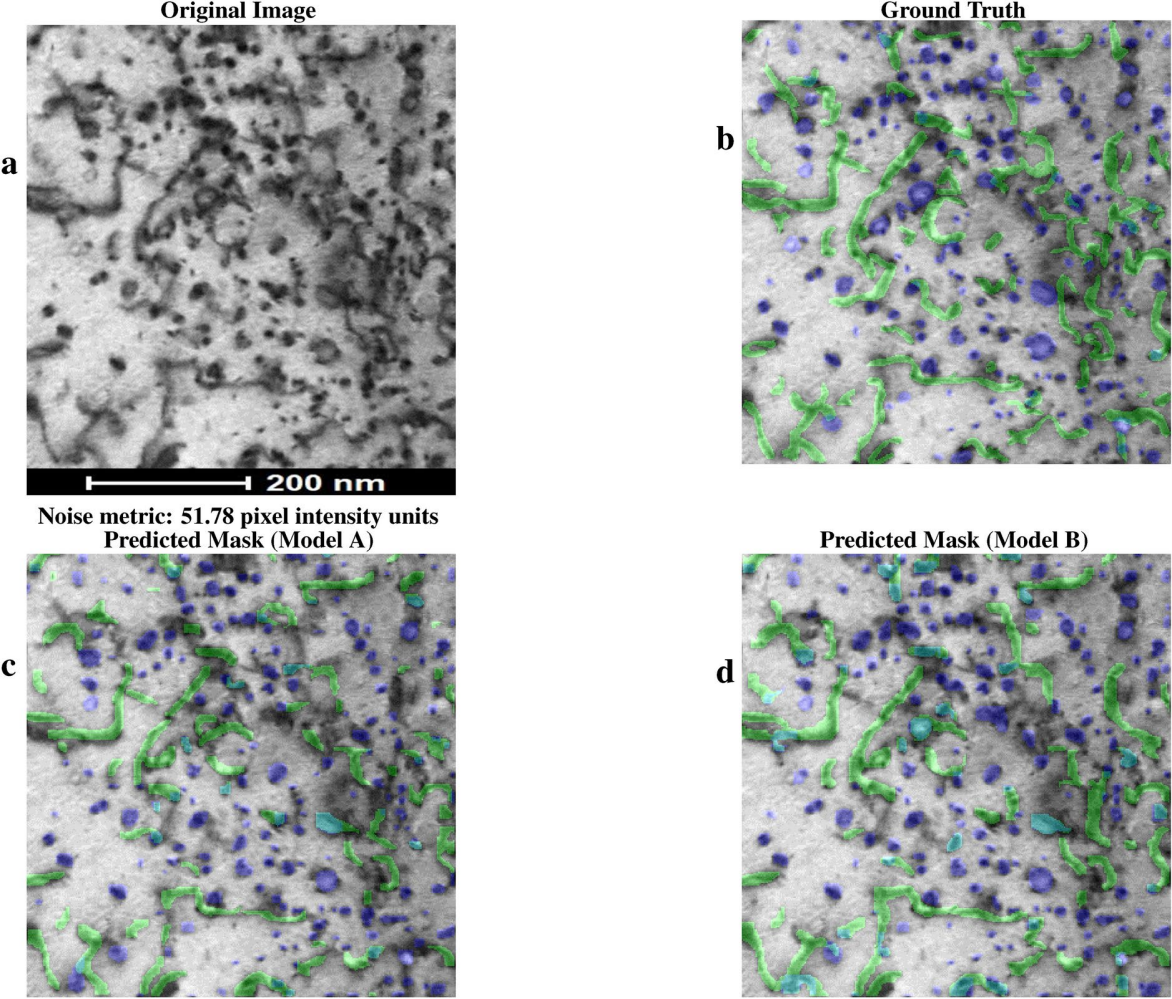
Comparison between the original image (**a**), ground truth labels (**b**), model A predictions (**c**), and model B predictions (**d**) of a BFSTEM image from the MA956 ODS alloy $\hbox {Fe}^{2+}$ irradiated to 50 dpa at $190^\circ$C under 111 zone axis. Green masks show line predictions, blue masks show loop predictions, and teal show overlapping predictions.

Dislocation loops and lines in MA956 ODS alloy images from the test dataset were predicted using model A and B and then compared with human-annotated ground truth masks. The ground truth masks and model inferences are shown in Figs. 2 and 3. Figure 2a shows an original BFSTEM image of the MA956 ODS alloy which contains both dislocation lines and loops. The image was taken under [001] zone axis where Fe^2+^ was irradiated to 25 dpa at 320°C. Ground truth human-annotated labels are added to the original image in Fig. 2b.

**Fig. 4:**
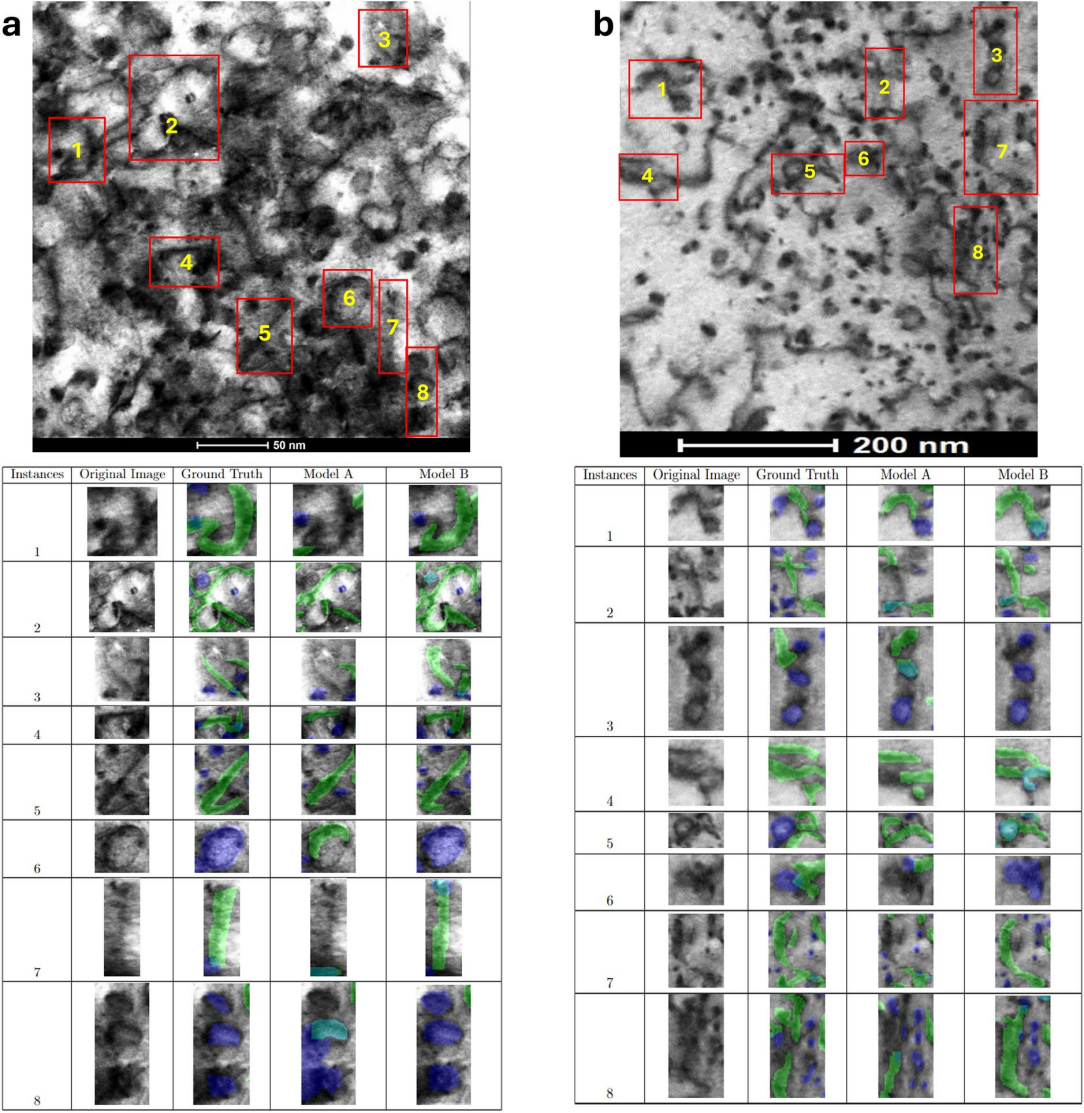
Comparison of model diagrams showing the differences in model prediction. (**a**) shows the comparison between model A and B prediction against the ground truth for Fig. 2. (**b**) shows the comparison between model A and B prediction against the ground truth for Fig. 3. Visual assessment shows model B has better prediction quality. Green masks represent lines, blue masks represent loops, and teal masks represent overlapping predictions.

Figure 2c overlays prediction masks inferred using model A, resulting in an F1 score with the ground truth of 0.39 for lines and 0.42 for loops. Similarly, Fig. 2d overlays masks inferred using model B, resulting in an F1 score with the ground truth of 0.57 for lines and 0.57 for loops. Figure 4a shows a zoomed in view of specific subregions of Fig. 2 with the ground truth and model inference masks presented for visual comparison. Calculated metrics and visual comparisons between models A and B in Figs. 2c, d, and 4a indicate that model B has a higher quality of identifications and localizations compared to model A.

Figure 3 is another irradiated MA956 ODS alloy BFSTEM image containing both dislocation lines and loops that was taken under [111] zone axis, Fe^2+^ irradiated to 50 dpa at 190°C. The original image is shown in Fig. 3a. The ground truth human-annotated labels were added to the original image and are shown in Fig. 3b. As in Fig. 2, dislocation defect prediction masks for model A are overlaid in Fig. 3c while those for model B are overlaid in Fig. 3d. For model A, the F1 score is 0.51 for lines and 0.55 for loops. For model B, the F1 score is 0.60 for lines and 0.59 for loops. Figure 4b shows a zoomed in view of specific subregions of Fig. 3 with the ground truth and model inference masks presented for visual comparison.

**Fig. 5:**
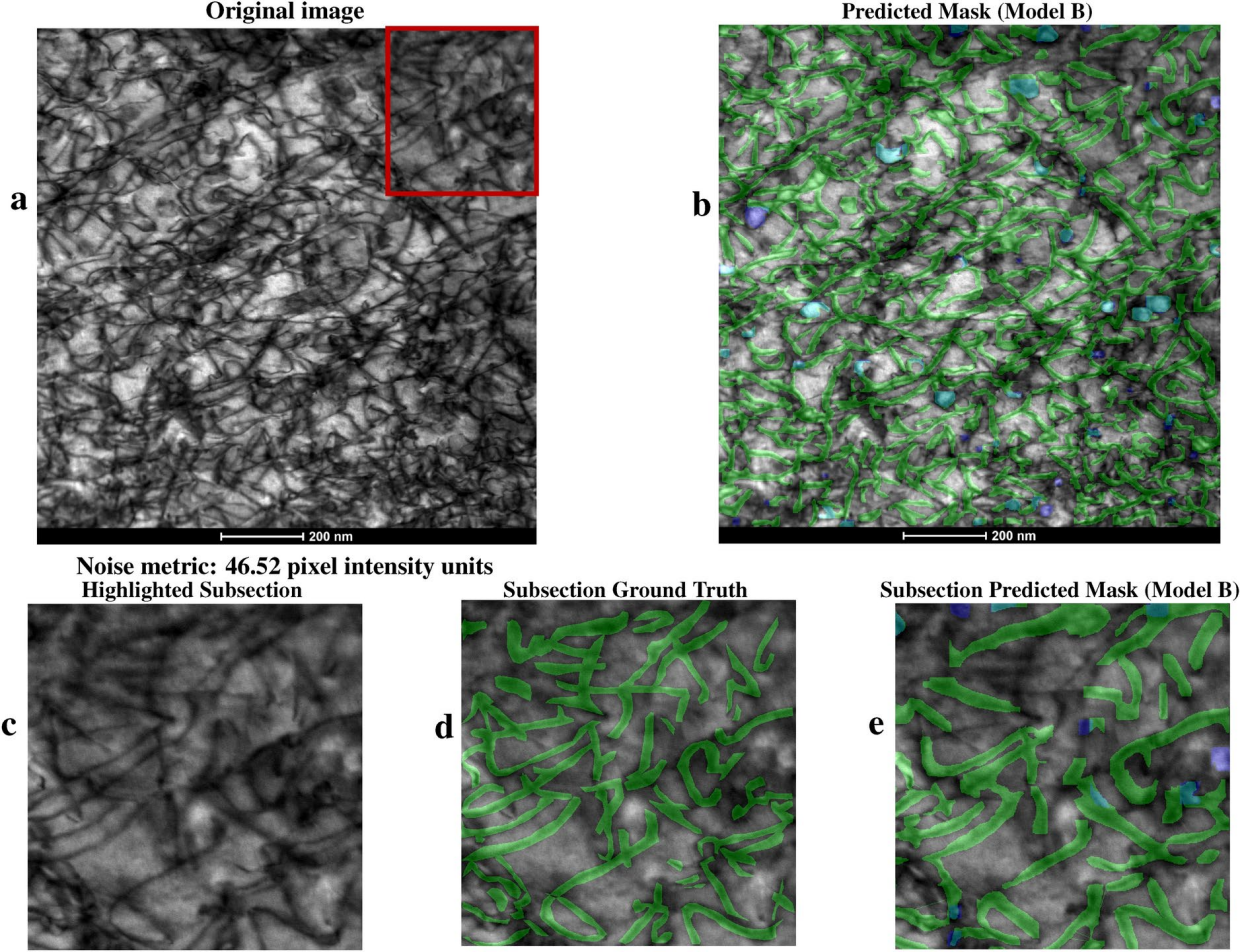
An HfAl alloy image with dislocation defects is shown in (**a**) and model B predictions on the image are shown in (**b**). Model B was not trained on HfAl images. Green masks represent predicted lines, blue masks represent predicted loops, and teal represents overlapping predictions. This image has complex overlapping line defects as well as substantial noise that makes human annotation very difficult while model B had no difficulty making visually compelling defect quantifications. To quantify this, a subsection from the full HfAl image in (**a**) is further analyzed in (**c**) through (**e**). The highlighted subsection of the full image is shown in (**c**), the ground truth annotations of that subsection are shown in (**d**), and the cropping of the predictions in (**b**) for the subsection are in (**e**). The F1 score for lines in this subsection is 0.59 which is consistent with results found for other alloys analyzed in this study – see Table 2.

Model B was also used to infer dislocation loops and lines on FeCrAl and HfAl alloy images, which are alloys not included in the training dataset. Figure 5 shows a HfAl TEM image with a partial human-annotated ground truth, where the predicted dislocation defects from model B are shown. The image contains multiple overlapping dislocation features and is noisy, but model B is able to identify and localize a substantial number of dislocation defects. A subsection of this image was human annotated to provide a ground truth comparison with the predictions of model B resulting in an F1 score of 0.59 for lines.

**Fig. 6:**
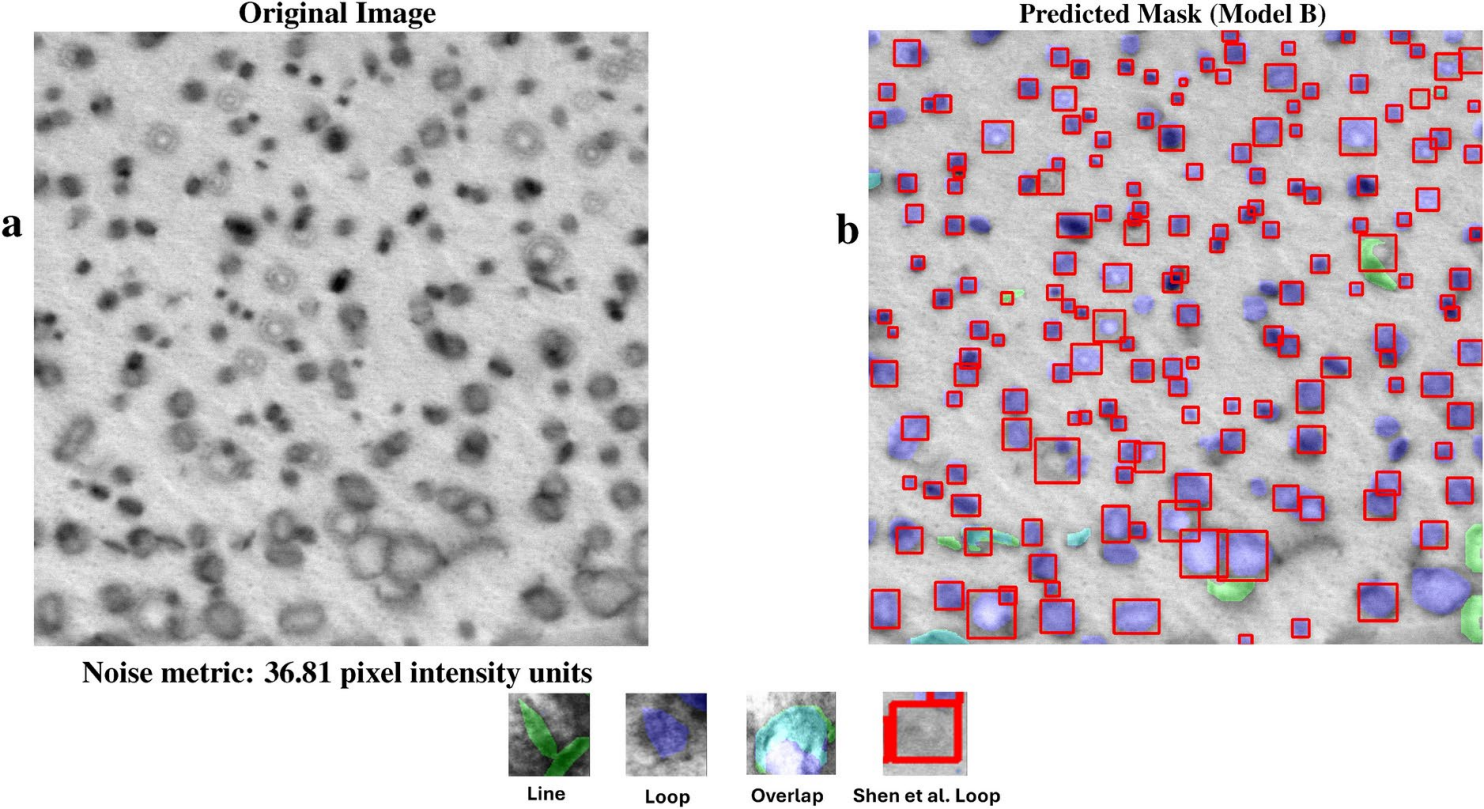
Diagram shows the original image of FeCrAl from Shen et al.^7^ in (**a**) and the model B predictions with ground truth bounding boxes in (**b**). Red boxes are ground truth labels by Shen et al., green masks show line predictions, blue masks show loop predictions, and teal masks show overlapping predictions.

Figure 6 shows an FeCrAl alloy image from Shen et al. that is dominated by dislocation loop defects, and is a low noise image with almost half the noise of Fig. 2 . The ground-truth human annotations for this test image are the bounding boxes provided by Shen et al.^7^ rather than polygon segmentations. The precision, recall, and F1 score for the dislocation loops are provided in Fig. 6b. The model B F1 score using the ground truth provided by Shen et al. is 0.70 for loops on Fig. 6b.

Inference speeds per image for model B were around 12.2 milliseconds per image on an NVIDIA A100 GPU. The high speed suggests wide scalability and time-saving potential for the materials science community in reproducibly quantifying dislocation defects, especially in in-situ experiments where the dislocation defects evolve with time.

**Table 2:** Summary of segmentation performance for models A and B. Model B substantially outperforms the control case, model A, in the test images.

Model used	Figure	Precision lines	Recall lines	F1 lines	Precision loops	Recall loops	F1 loops
A	2c	0.50	0.32	0.39	0.53	0.35	0.42
B	2d	0.56	0.58	0.57	0.67	0.50	0.57
A	3c	0.61	0.44	0.51	0.55	0.55	0.55
B	3d	0.60	0.58	0.60	0.52	0.67	0.59
B	5e	0.56	0.61	0.59	N/A	N/A	N/A
B	6b	N/A	N/A	N/A	0.70	0.70	0.70

**Fig. 7:**
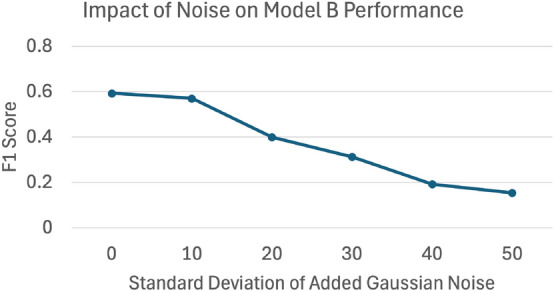
In this Figure, the F1 score for lines from model B is plotted as a function of artificial Gaussian noise added to the image in Figure 3. The artificially added noise was blended with the image where the contrast ratio was normalized.

## Discussion

The ability for the transfer model learning strategy illustrated by model B to predict on both clear and blurry STEM images allows for real-life TEM image analysis. Figure 7 shows the relationship between increasing noise in an image and the resulting F1 Score of model B’s predictions. The ground truth image from Figure 3 had varying standard deviations of pixel intensities worth of Gaussian noise blended in and then normalized for consistent contrast ratios, which were then predicted on by model B. Using the pixel noise level as a benchmark, the images in Figs. 2, 3, and  5 are significantly more noisy than images tested in similar machine learning efforts at dislocation defect quantification. For example, in Shen et al.^7^, the test dataset had an average noise of 35.91 pixel intensity units between twelve images. In contrast, this study’s models were intentionally tested on quantifying dislocation defects in high noise images ranging from 46.52 to 67.28 pixel intensity units. Additionally, this work also explored a low noise test image in both Li et al.^6^ and Shen et al.^7^ to compare F1 scores for a low noise image. For Figure 6, the F1 score from model B was 0.70 where the average F1 score for the test images reported from Shen et al. is 0.78. While these results are comparable, it is noted that models A and B in this work were trained to identify and segment both disclocation lines and loops simultaneously whereas this was not the case in other similar studies^6–8^.

During testing, model B has been found to predict accurately on alloys beyond the alloy in the training dataset (MA956 ODS), such as HfAl and FeCrAl. By effectively predicting on images of different qualities and alloy compositions with no post-processing, model B’s predictive capabilities of dislocation lines and loops can assist scientists trying to quantify defects with minimal TEM defect annotated training images.

This study only used five TEM defect annotated images that were augmented to 45 for use in training, which is fewer TEM defect training images compared to most contemporary models. Because of the time complexity required for human annotation of TEM micrographs, the lack of training data in dislocation defect machine learning approaches is expected to be persistent. However, the improvement in results of model B over model A shows the vital role of topic-adjacent datasets for use in transfer learning to improve defect quantification results.

The transfer learning strategy in this study uses on average fewer epochs compared to contemporary models, which should result in noticeable time savings during model training. For example, in this study, model A was trained for 100 epochs, the crops and cavities model was trained for 200 epochs, and model B was trained for 100 epochs. On an NVIDIA A100 40 GB GPU, model B finishes training in roughly 30 minutes. For comparison, the YOLOv3 approach^7^ for dislocation defects required 18,300 epochs for training. The transfer learning strategy of model B significantly reduces the training time required on the human-annotated TEM micrographs.

## Conclusion

This study describes a transfer learning strategy with YOLO11 for the identification and localization of dislocation line and loop defects via segmentation in post-irradiation examination. The approach is particularly effective when working with a very small training dataset containing images with varying degrees of blurriness and ambiguous features. Dislocation defect quantification was demonstrated on three materials relevant to nuclear energy applications, including two alloys that were not represented in the training dataset. The ability to identify and localize dislocation loops and lines in micrographs with different qualities and alloy compositions, with no post-processing using a standard open-source computer vision model, significantly lowers the barrier to fast and reproducible material characterization and analysis.

Because the MA956 ODS alloy has relatively large oxide particles, future work will involve studying another ODS alloy with finer oxide particles, where the oxide particle size is similar to dislocation loops. As part of future work, it is intended to train the model with both BFSTEM and High-Angle Annular Dark-Field STEM images acquired simultaneously so that the model can distinguish oxide particles from dislocation loops.

## Data Availability

The datasets generated and/or analysed during the current study are available from the corresponding author (matthew.anderson2@inl.gov) on reasonable request.
